# Supplementary material for: Androgen deprivation promotes neuroendocrine differentiation and angiogenesis through CREB-EZH2-TSP1 pathway in prostate cancers
Source: Nat Commun. 2018 Oct 4;9:4080. doi: 10.1038/s41467-018-06177-2 (PMC6172226; doi:10.1038/s41467-018-06177-2)
Supplement: Supplementary file 1 — Supplementary Information [file 41467_2018_6177_MOESM1_ESM.pdf]

Supplementary Information for

Androgen deprivation promotes neuroendocrine differentiation and angiogenesis through CREB-EZH2-TSP1 pathway in prostate cancers

Y Zhang, *et al*.

p-CREB	H3K27me3		
	+	++	+++
+	13	13	4
++	3	15	3
+++	3	12	12

Supplementary Table1. Results of IHC staining of tissue microarrays with 78 cases of human prostate cancer and normal samples. Chi-square test showed that p-CREB levels positively correlate with the levels of H3K27me3 (Chi Square $\chi^2 = 16.4$, $P = 0.003$)

Supplementary Table2. Information of Antibodies

Antibody name	manufacturer	Catalog #	Dilution
**Actin**	Cell Signaling Technology	4967L	1:1000
**CREB**	Millipore Corp	06-863	1:1000
**CHGA**	Abcam	ab15160	1:100
**ENO2**	LSBio	LS-C62804	1:500
**EZH2**	Cell Signaling Technology	5246S	1:1000
**GAPDH**	Cell Signaling Technology	2118S	1:1000
**H3**	Cell Signaling Technology	9783T	1:1000
**H3K27me3**	Cell Signaling Technology	4909T	1:1000
**p-CREB**	Cell Signaling Technology	9198S	1:1000
**SYP**	Cell Signaling Technology	5461T	1:1000
**TUBB3**	Genscript	89494-648	1:1000
**TSP1**	Abcam	ab85762	1:500
**CD31**	Abcam	ab28364	1:500

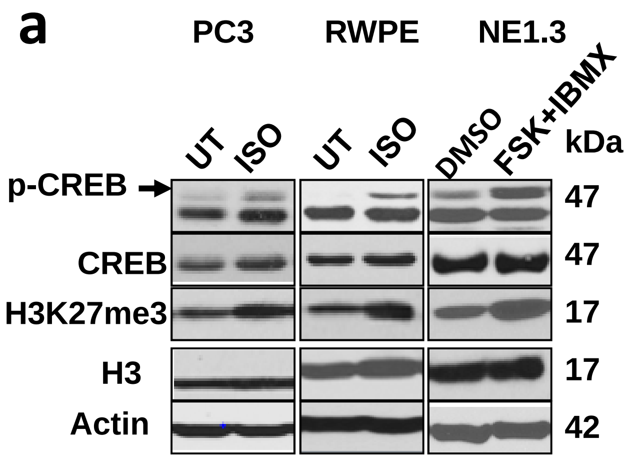

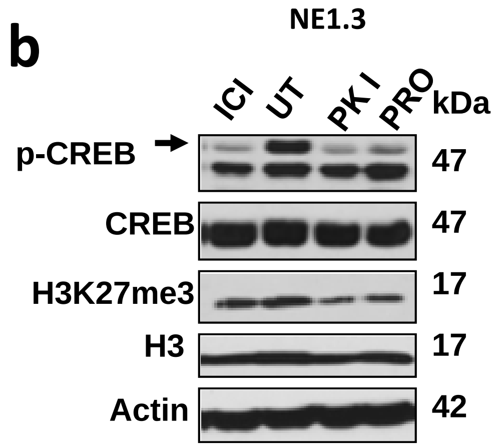

Supplementary Figure 1. ADT activates EZH2 through PKA/CREB signaling.

(a) PC3 and RWPE cells were treated with 10 μM isoproterenol (ISO, β-adrenergic receptor agonist) for 24 hrs. NE1.3 cells were treated with 10 μM forskolin (FSK, adenylyl cyclase activator) + 0.5 mM IBMX (phosphodiesterase inhibitor) for 24 hrs. Western blots show that pS133-CREB and H3K27me3 levels were significantly upregulated upon activation of PKA/CREB signaling. **(b)** Treatments with PKA inhibitor PKI, beta-adrenergic antagonists ICI and propranolol (PRO) (all 10 μM, 48 hrs) significantly reduced pS133-CREB and H3K27me3 levels in NEPC NE1.3 cells. The figure is supplementary to Fig. 3.

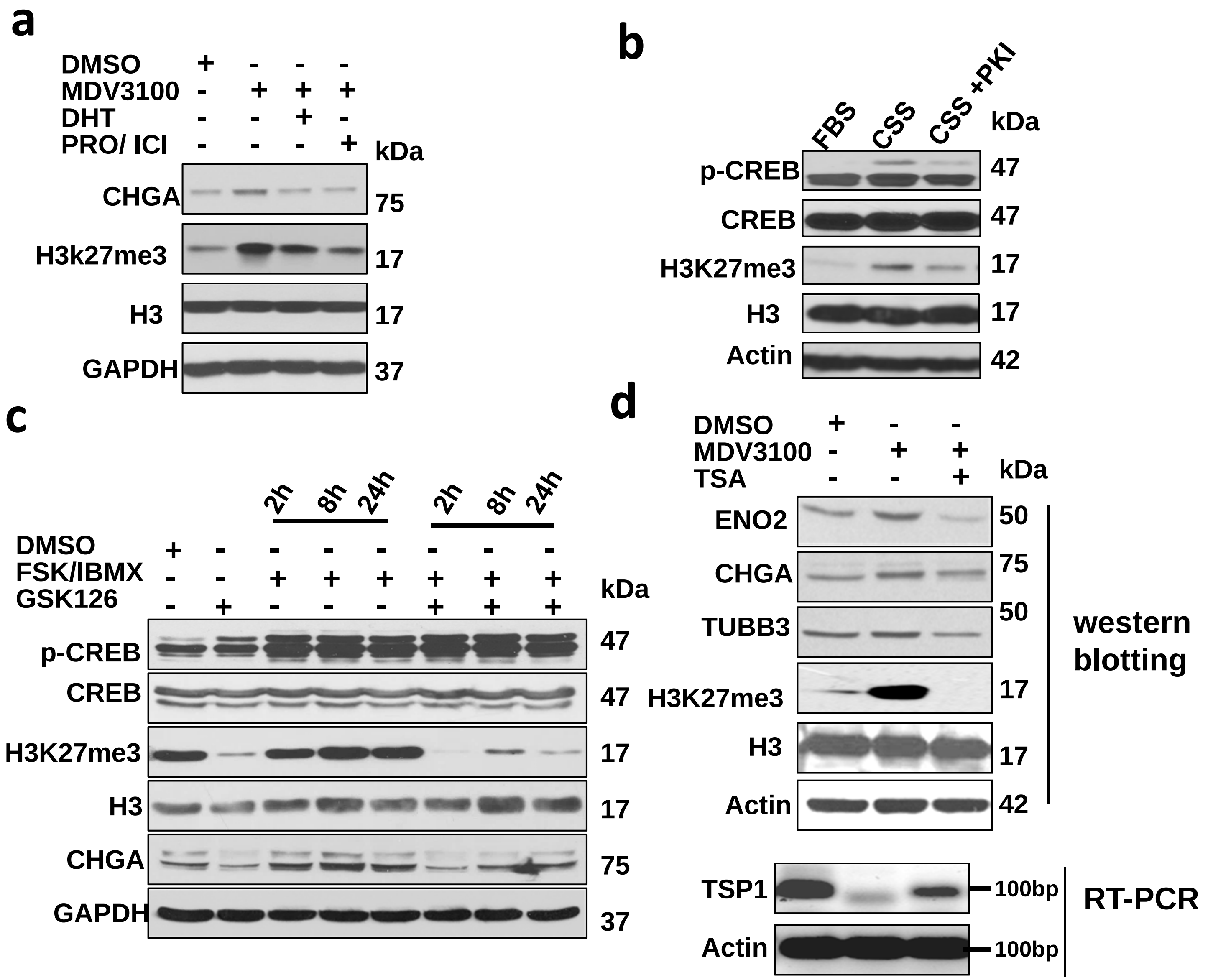

Supplementary Figure 2. ADT or PKA/CREB signaling activators in AR-positive cells activates the CREB/EZH2 axis and induces NE marker expression, which is reversed by DHT, inhibitors for CREB signaling or EZH2 partner HDACs.

(a) 22Rv1 cells were treated with 10 μM MDV3100, without or with 5 nM DHT or 10 μM PKA/CREB signaling inhibitor PRO+ICI. Expression levels of indicated proteins were analyzed by western blotting. **(b)** Western blotting for p-CREB and H3K27me3 in LNCaP cells growing in regular FBS, CSS (48 hr), CSS (48 hr) + PKA/CREB signaling inhibitor PKI (10 μM, 5 hr). **(c)** LNCaP cells were treated by 5 μM of EZH2 inhibitor GSK126 alone, CREB signaling activators 10 μM FSK+0.5 mM IBMX without or with 5 μM GSK126 for 2, 8 and 24 hr. Western blotting results for p-CREB, H3K27me3 and NE marker CHGA, along with loading controls, were shown. **(d)** Induction of H3K27me3 and NE markers (western blotting) and repression of TSP1 (RT-PCR) by MDV3100 treatment (10 μM, 72 hr) were reversed by HDAC inhibitor TSA (25 ng ml^{-1}, 72 hr).

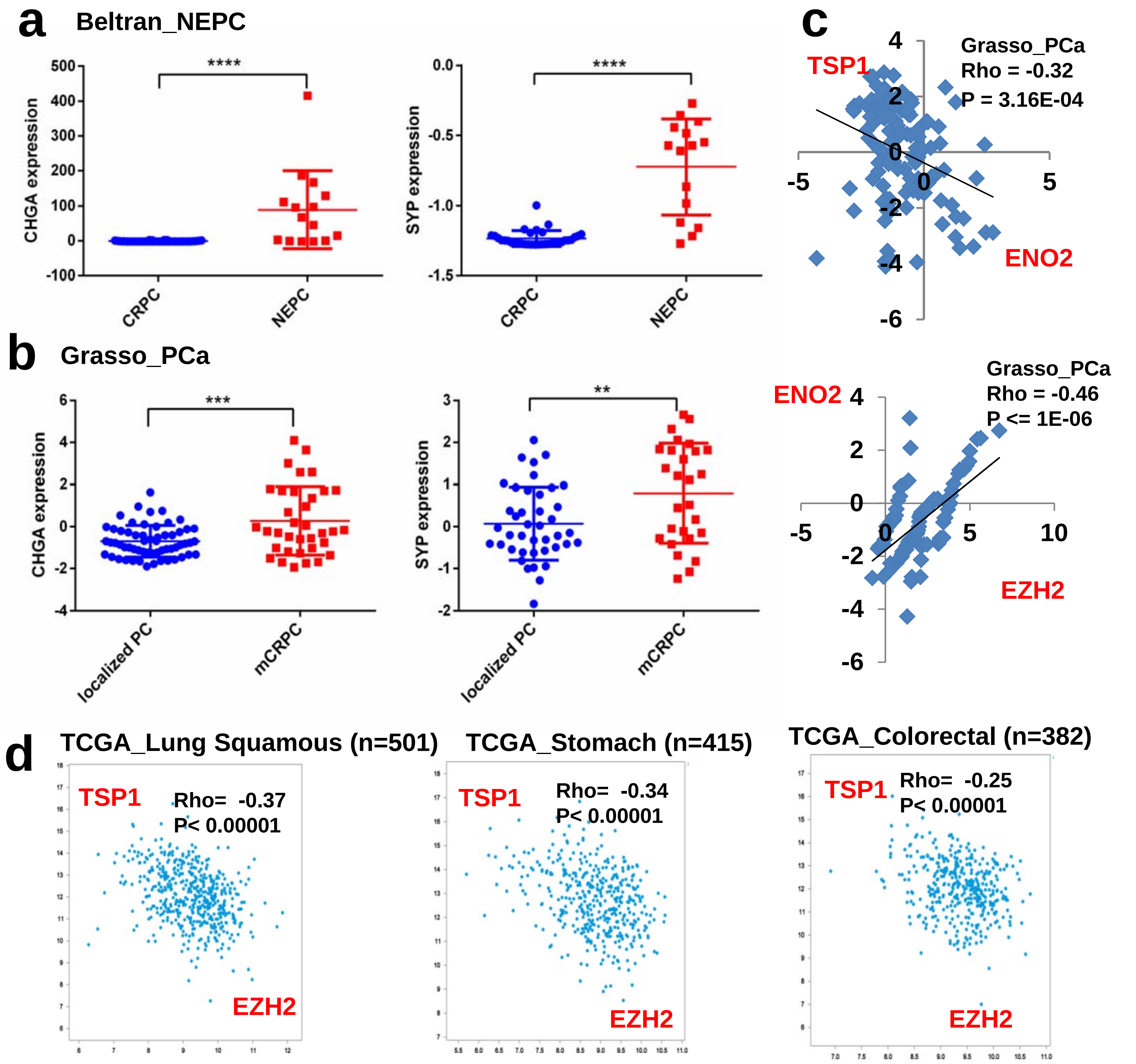

Supplementary Figure 3. Expression of NE markers in human prostate cancers progression and their correlation with the expression of TSP1 and EZH2.

(a) NE markers CHGA and SYP are expressed higher in metastatic mCRPCs than in localized tumors (Grasso_PCa). **(b)** NE markers CHGA and SYP are expressed higher in NEPC than in CRPC-adenocarcinoma (Beltran_NEPC). **(c)** NE marker ENO2 is negatively and positively correlated with TSP1 and EZH2, respectively, in mCRPC (Grasso_PCa). **(d)** TSP1 expression negatively correlates with EZH2 expression in large TCGA datasets for several common solid cancer types. Spearman correlation coefficient Rho and P values were indicated. TCGA datasets were accessed through the cBioPortal interface in May 2018. Scatter plots were downloaded directly from cBioPortal.org website. The figure is supplementary to Fig. 6.***P<0.0001; **<0.001.

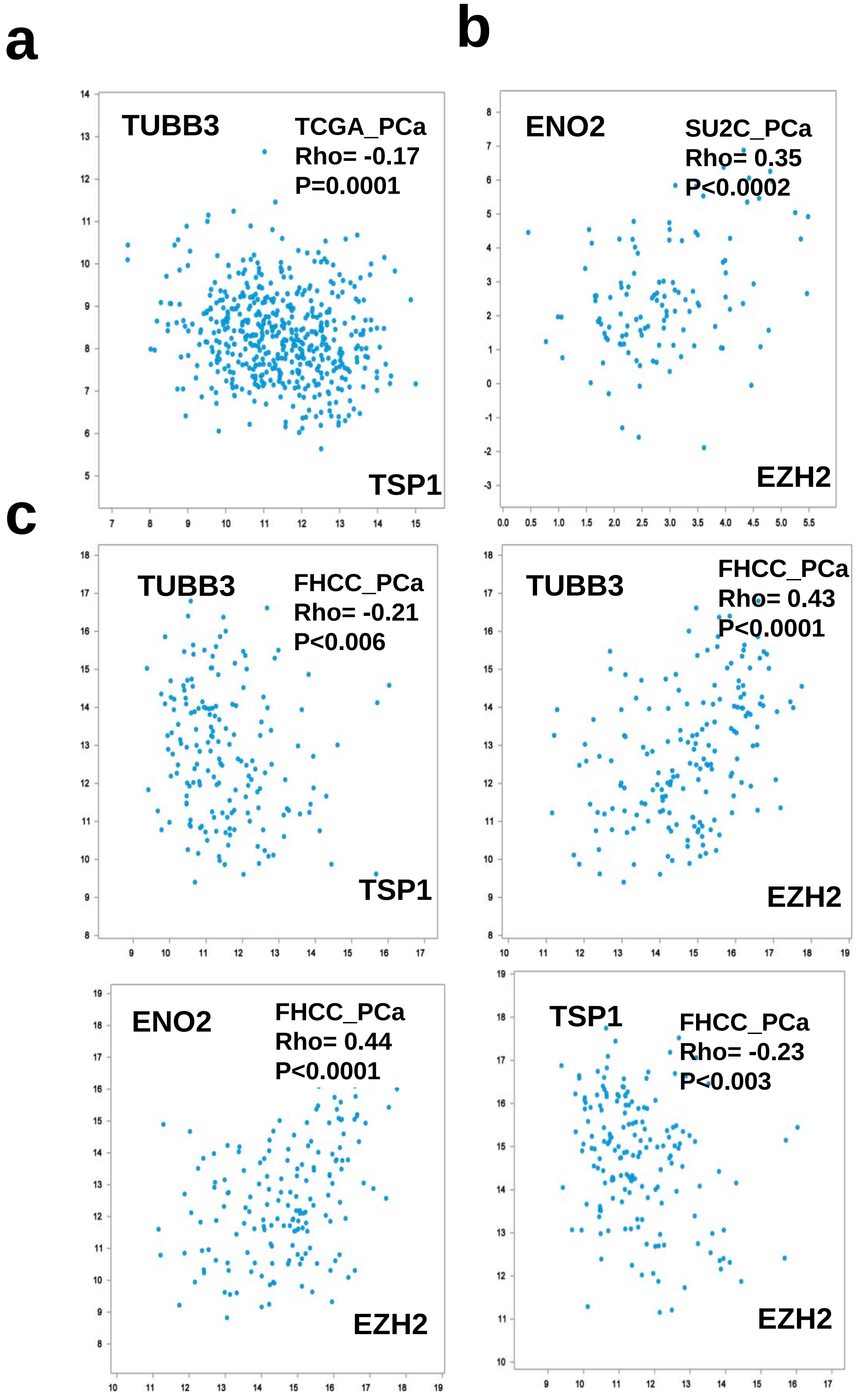

Supplementary Figure 4. Correlation of TSP1, EZH2 and NE markers ENO2 and TUBB3 in human prostate cancer datasets.

(a) TCGA primary prostate tumors (n=498), **(b)** SU2C mCRPCs (n=118), **(c)** Fred Hutchinson Cancer Research Center mCRPC (n=171). Spearman correlation coefficient Rho and P values were indicated. These datasets were accessed through the cBioPortal interface in May 2018. Scatter plots were downloaded directly from cBioPortal.org website. This figure is supplementary to Fig. 6.

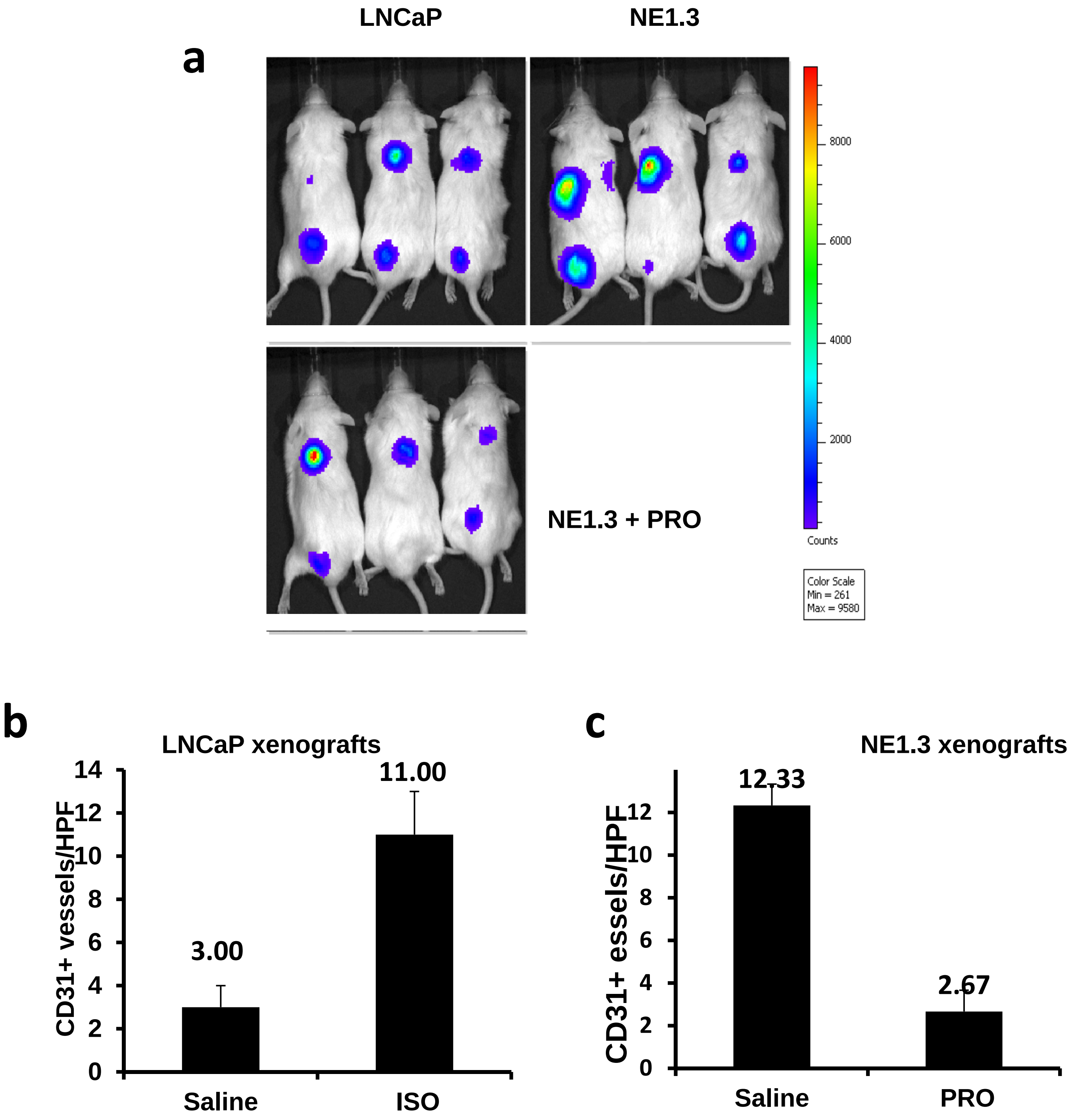

Supplementary Figure 5. LNCaP and NE1.3 cell-derived xenograft (CDX) tumors and quantification of angiogenesis in CDX tumors.

The figure is supplementary to **Fig. 10**. **(a)** Representative BLI images of mice in the three indicated groups at day 25. **(b)** The numbers of CD31+ microvessels per high-power field in sections of at least three different LNCaP tumors from either saline or ISO treated mice were counted, then averaged and plotted on Y-axis. **(c)** The numbers of CD31+ microvessels per high-power field in sections of at least three different NE1.3 tumors from either saline or PRO treated mice were counted, then averaged and plotted on Y-axis. Error bar represents mean ± SD.

Fig. 1a: LNCaP

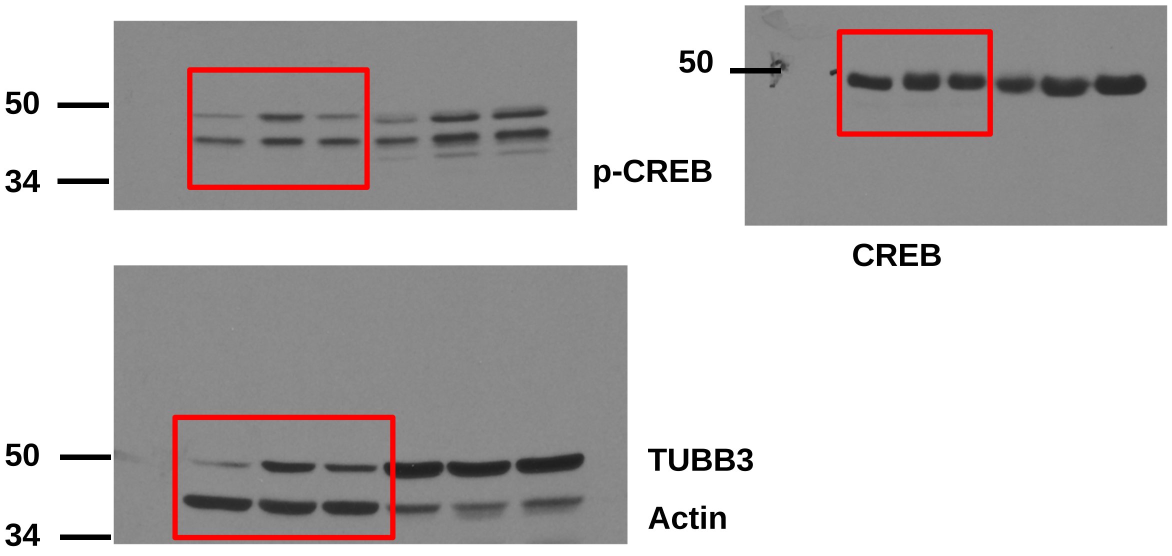

Fig. 1a: VCaP

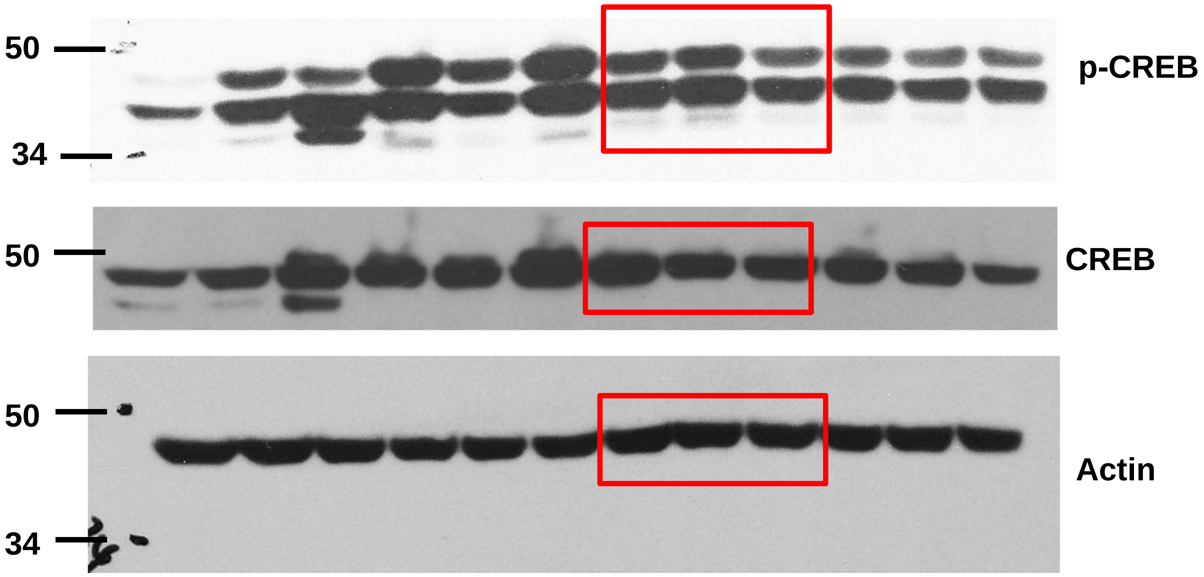

Fig. 1b

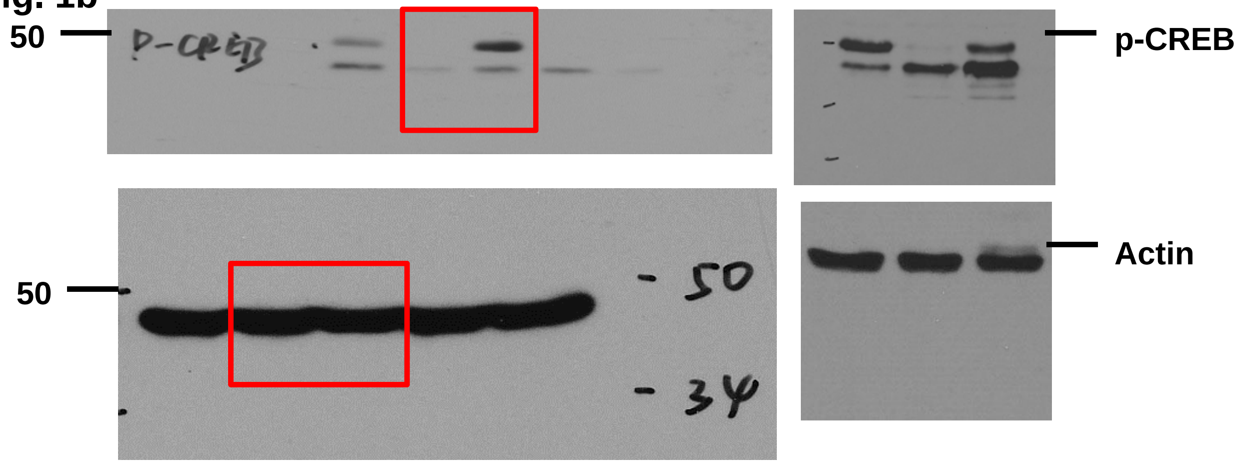

Fig. 1e: LNCaP-shCREB

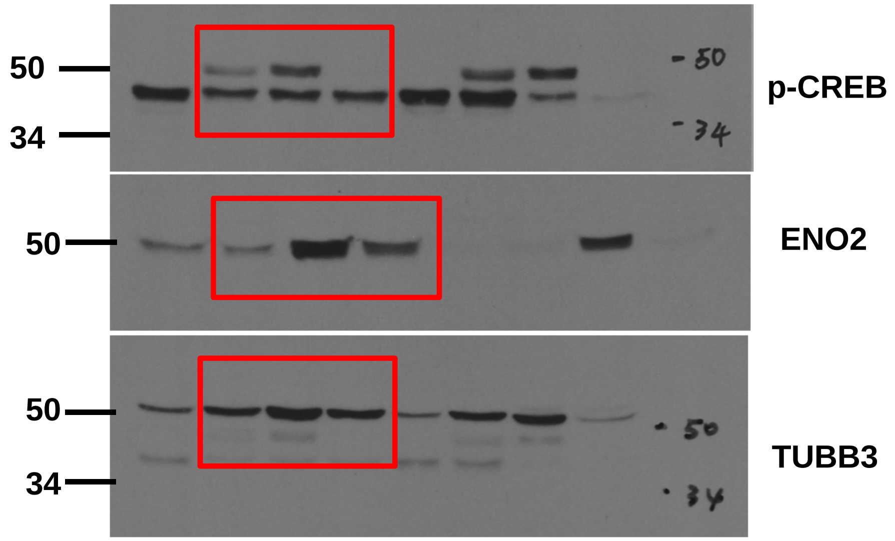

Fig. 1f: LNCaP-ACREB

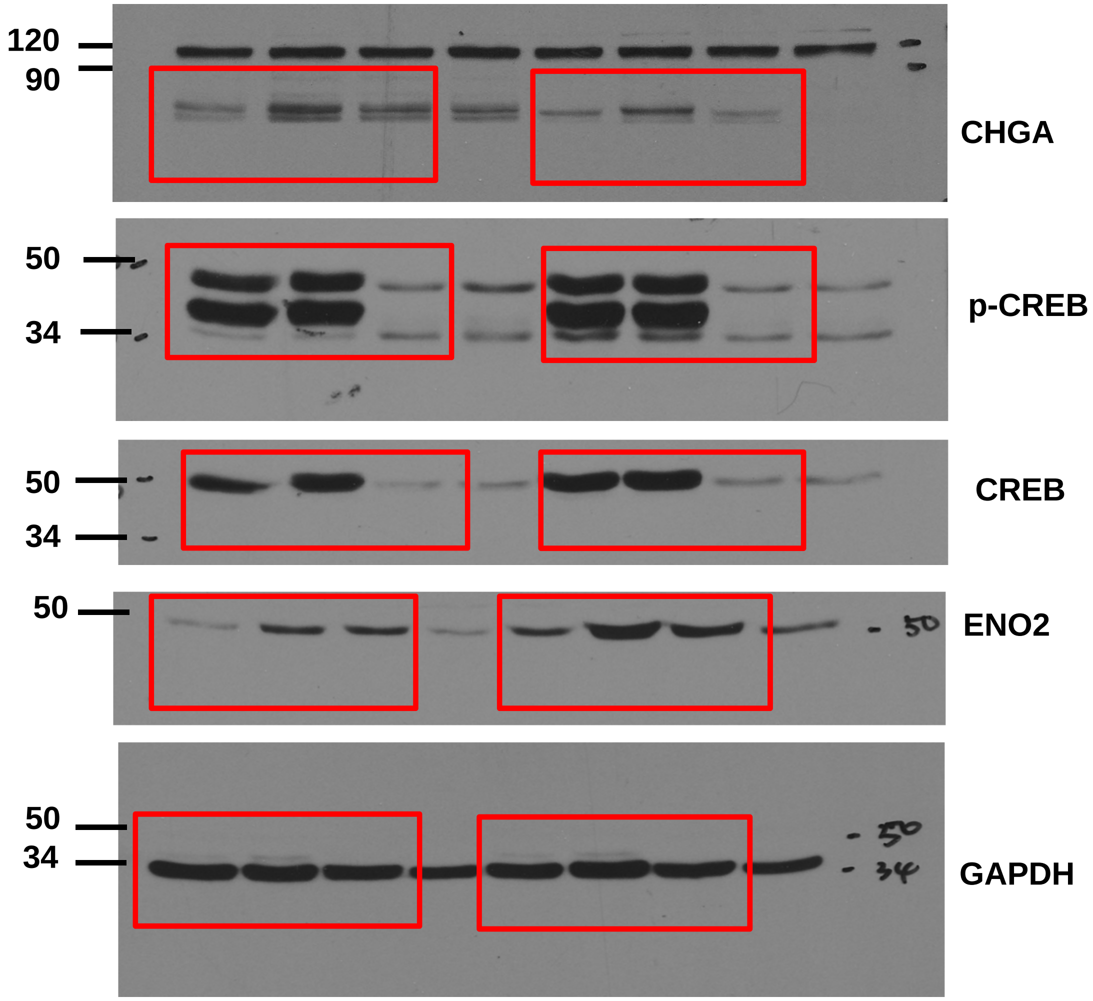

Fig. 2a: LNCaP

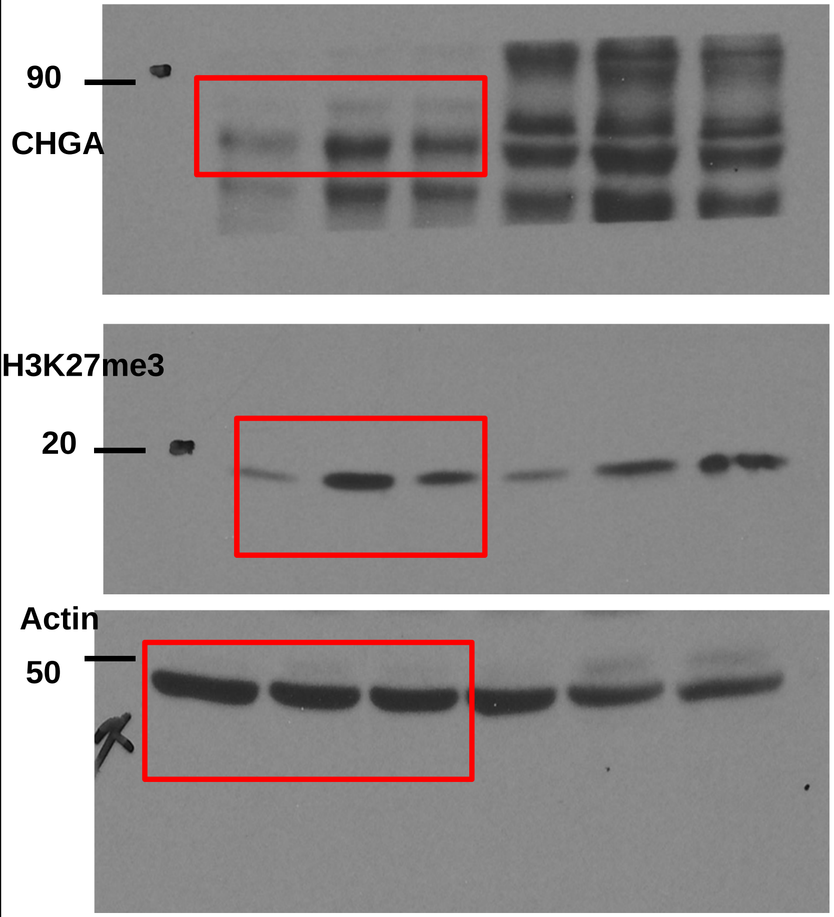

Fig. 2b:

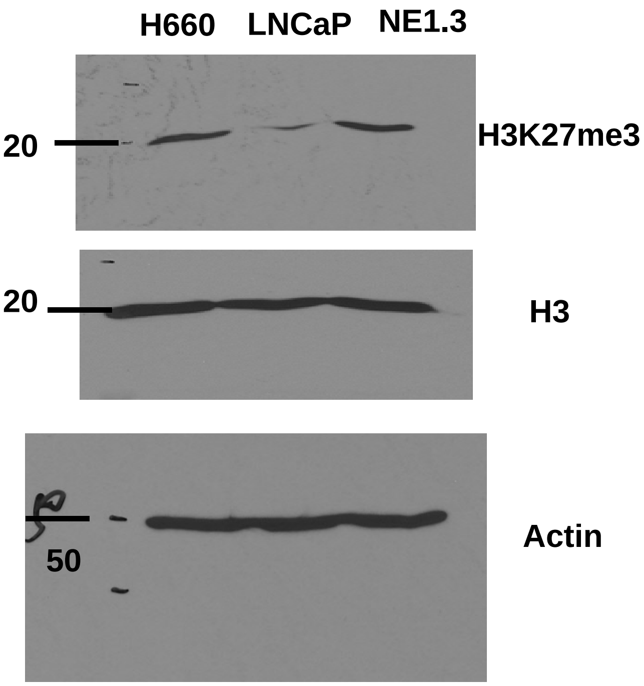

Fig. 2a: 22RV1

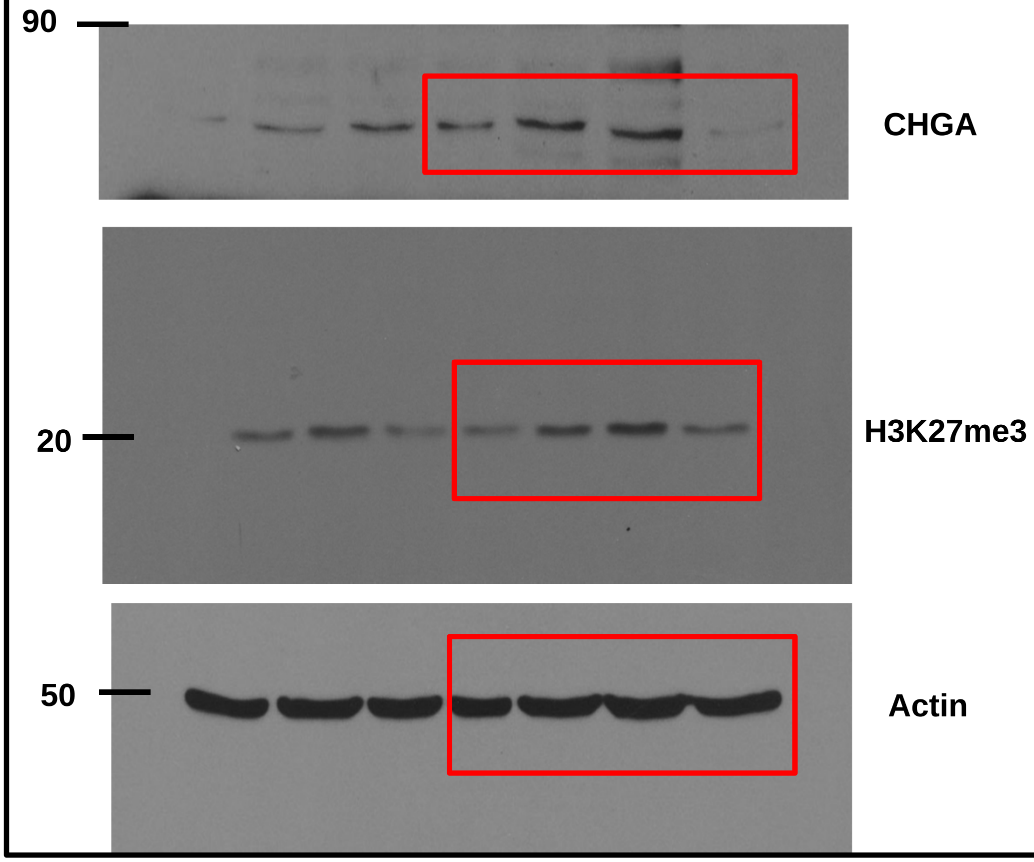

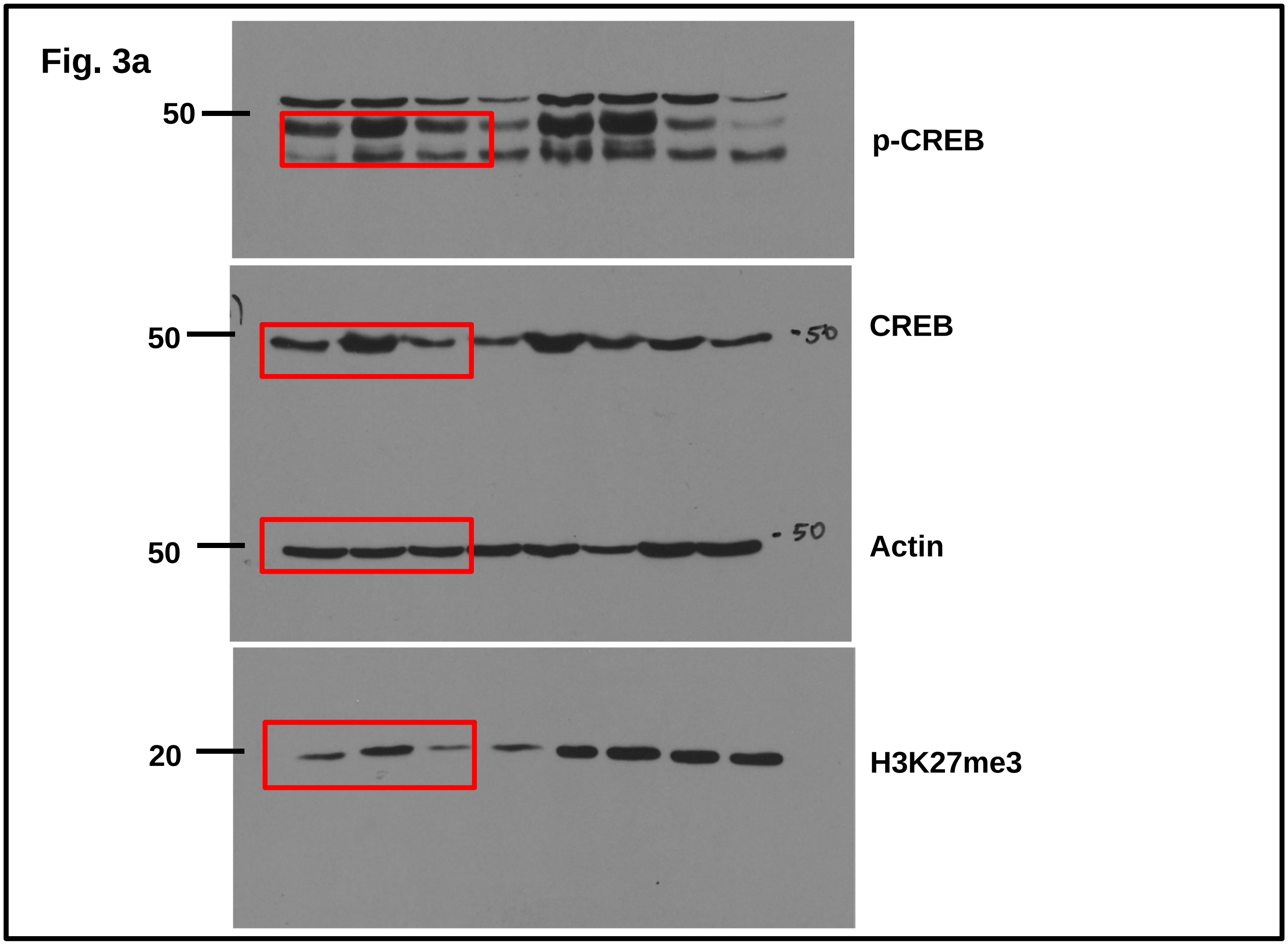

Fig. 3a
50
p-CREB
50
CREB
50
Actin
20
H3K27me3

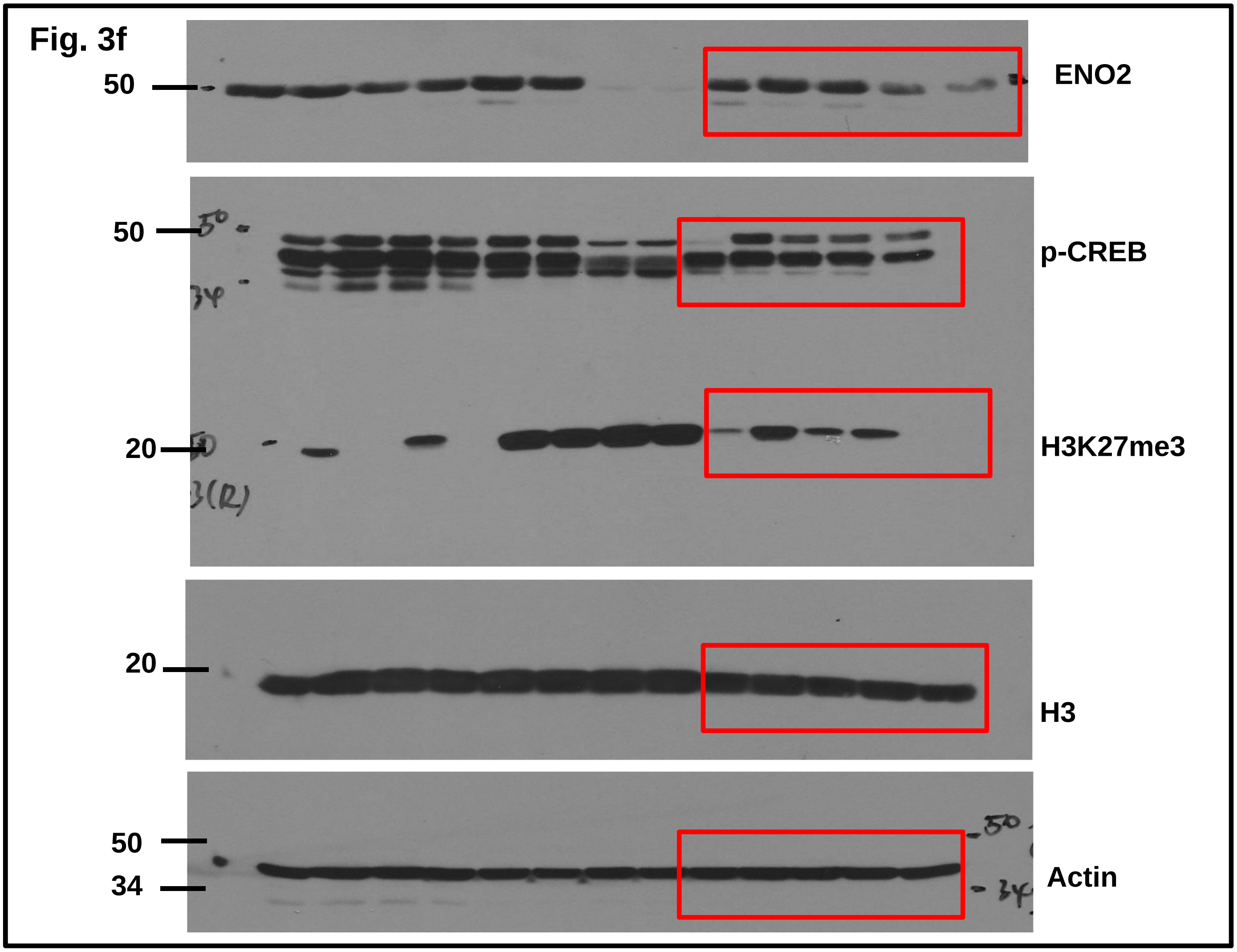

Fig. 3f
50
ENO2
50
p-CREB
20
H3K27me3
20
H3
50
34
Actin

Fig. 3c: 144 cell

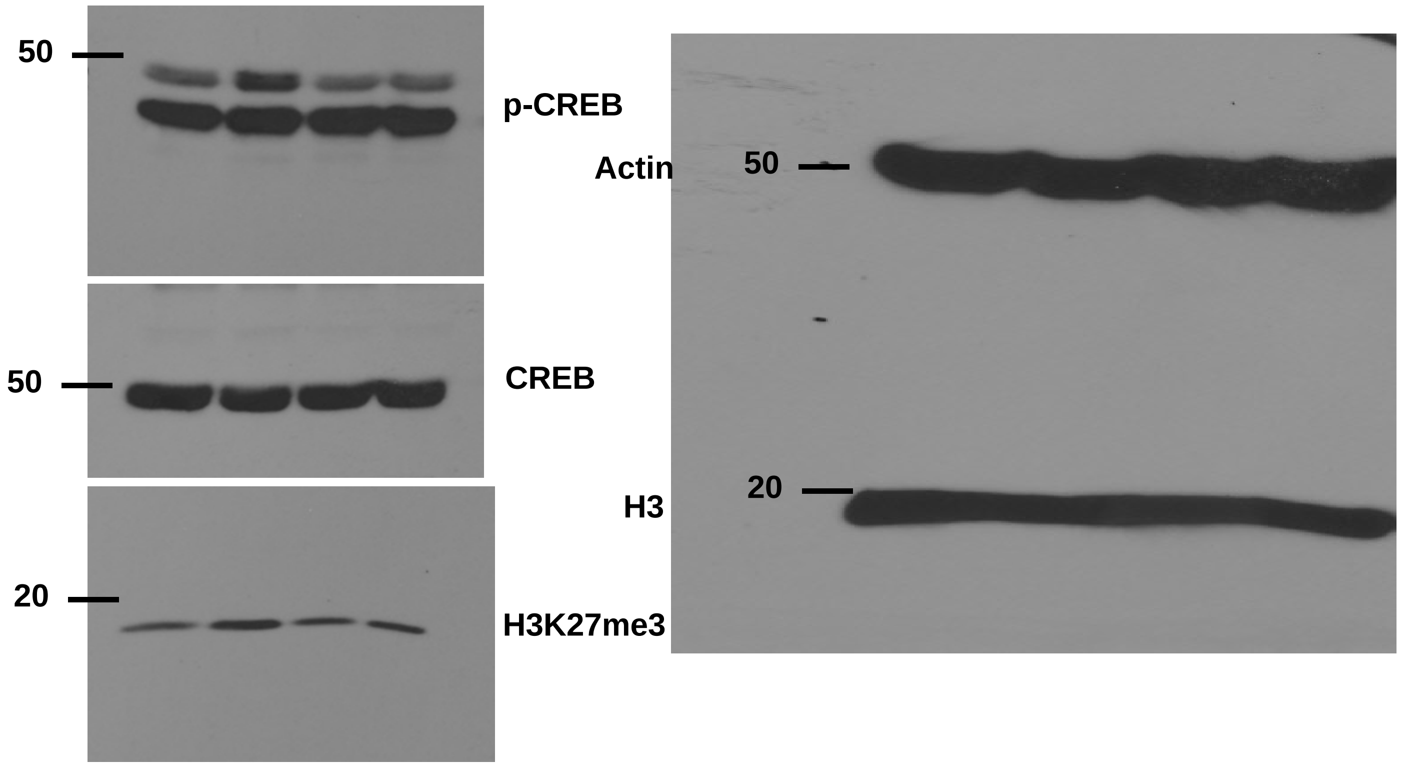

Fig 4b

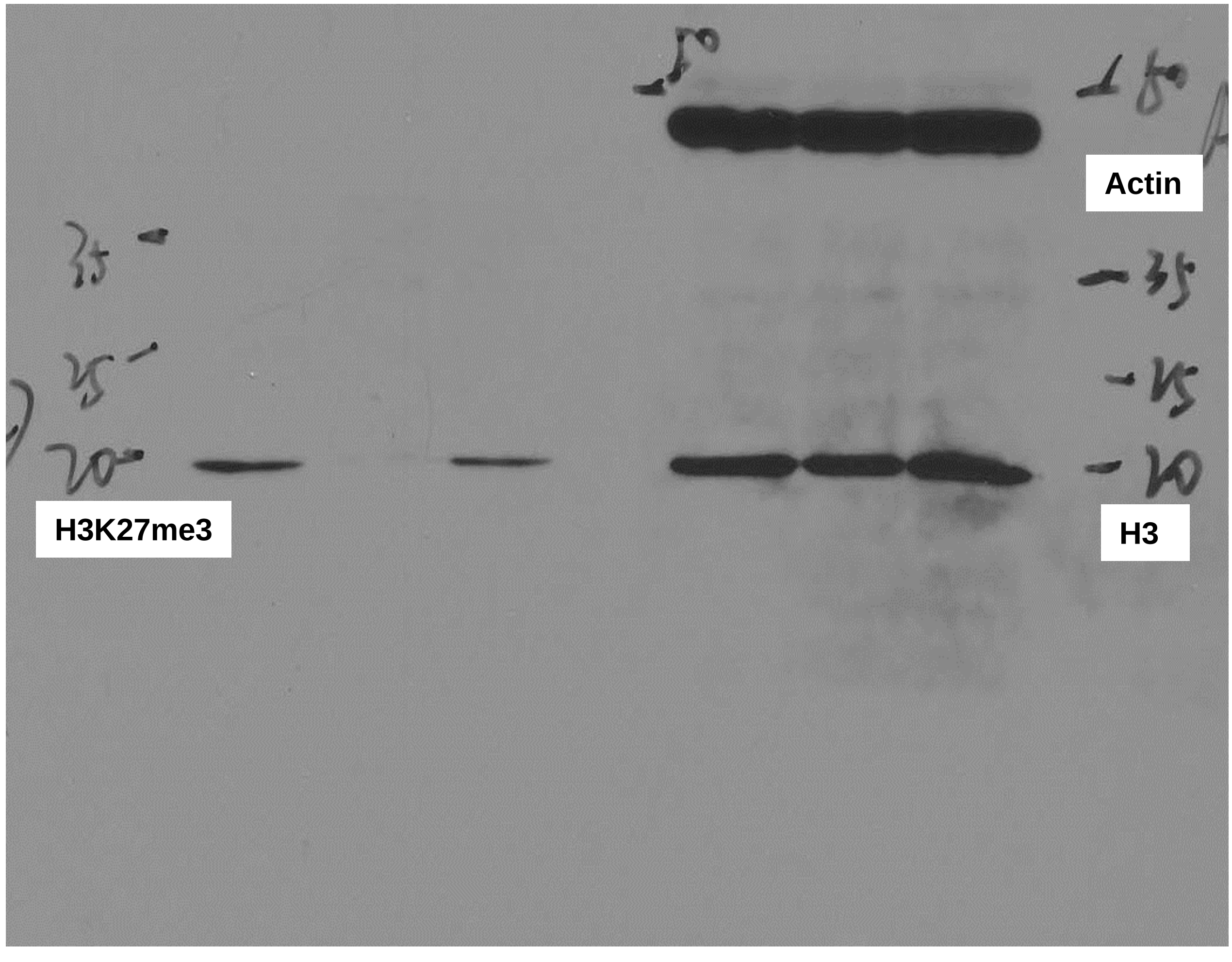

Fig. 4d

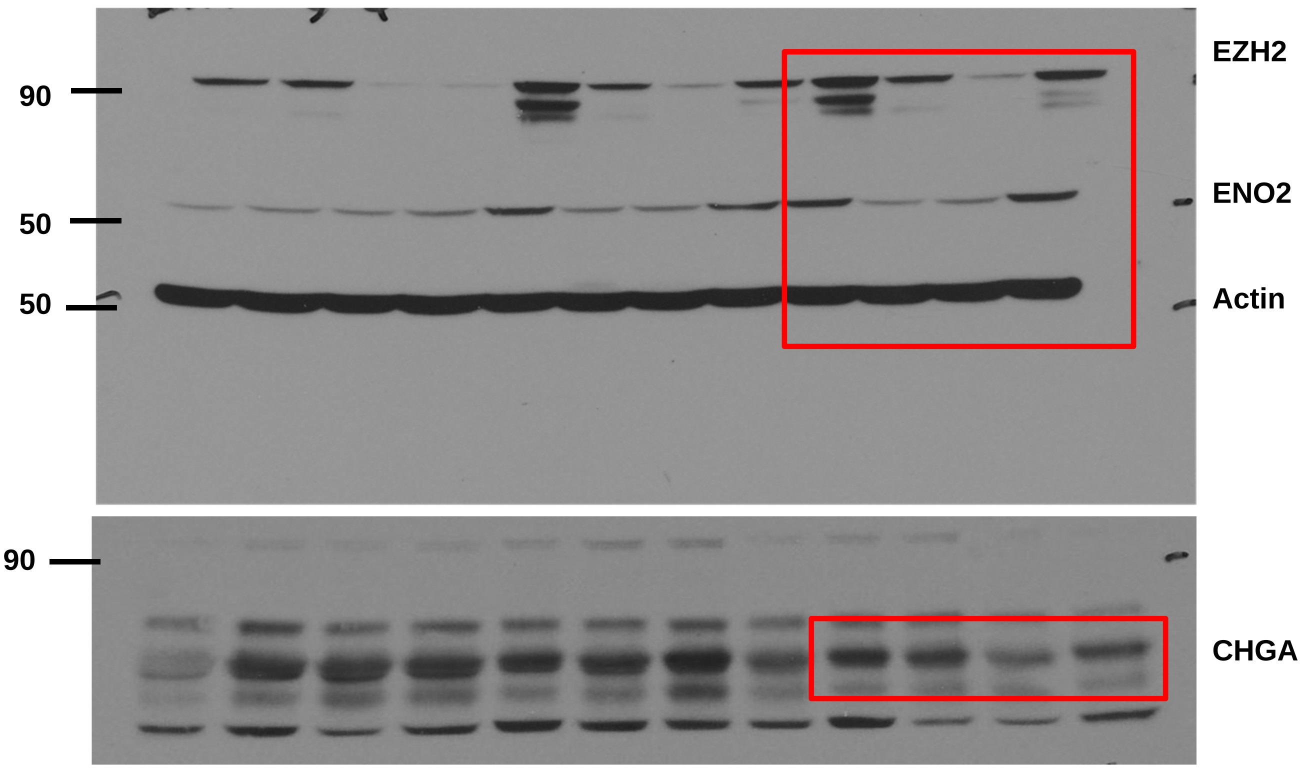
90
50
50
90
EZH2
ENO2
Actin
CHGA

Fig. 5b

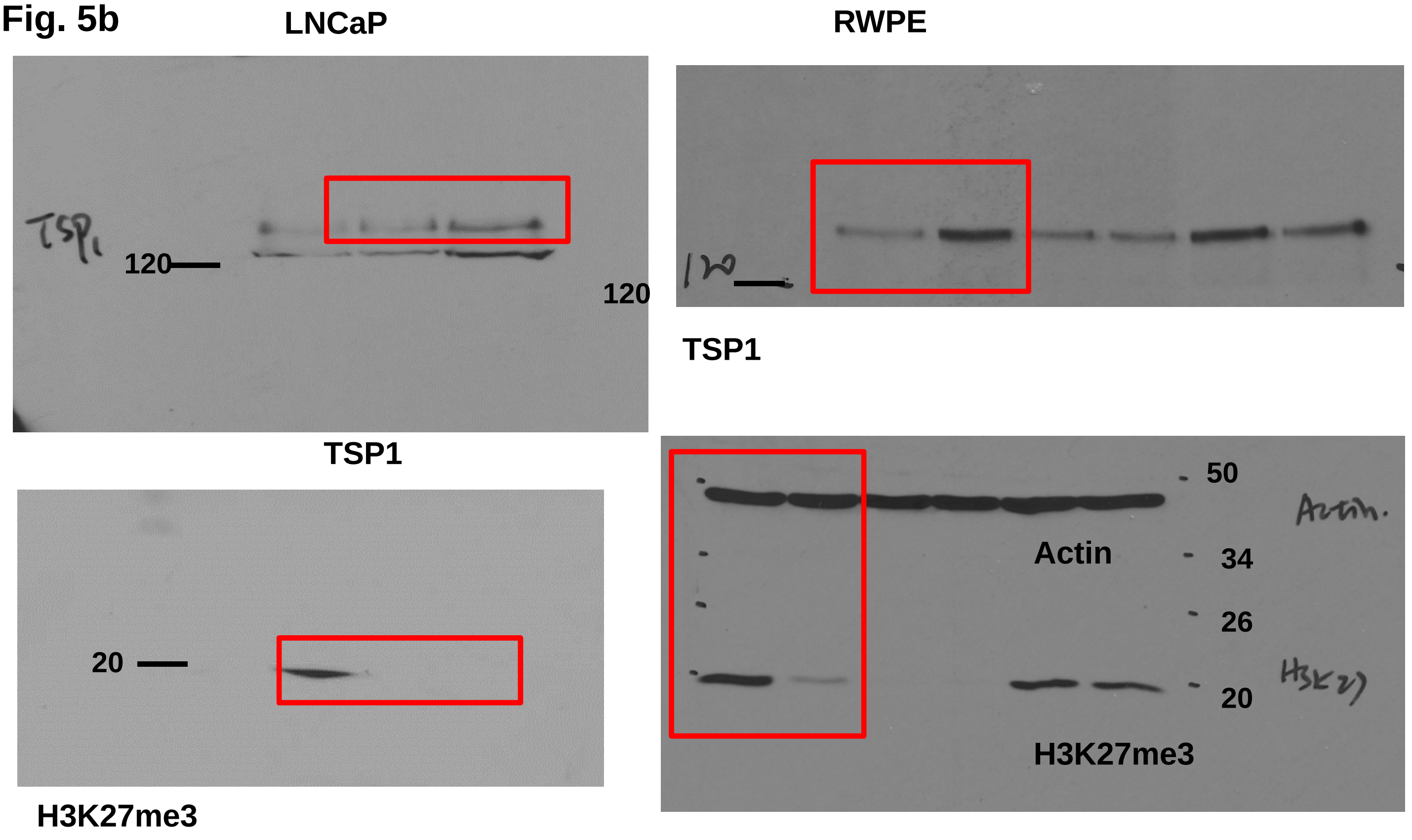
LNCaP
RWPE
120
120
TSP1
TSP1
20
H3K27me3
50
34
26
20
Actin
H3K27me3

Fig. 5c

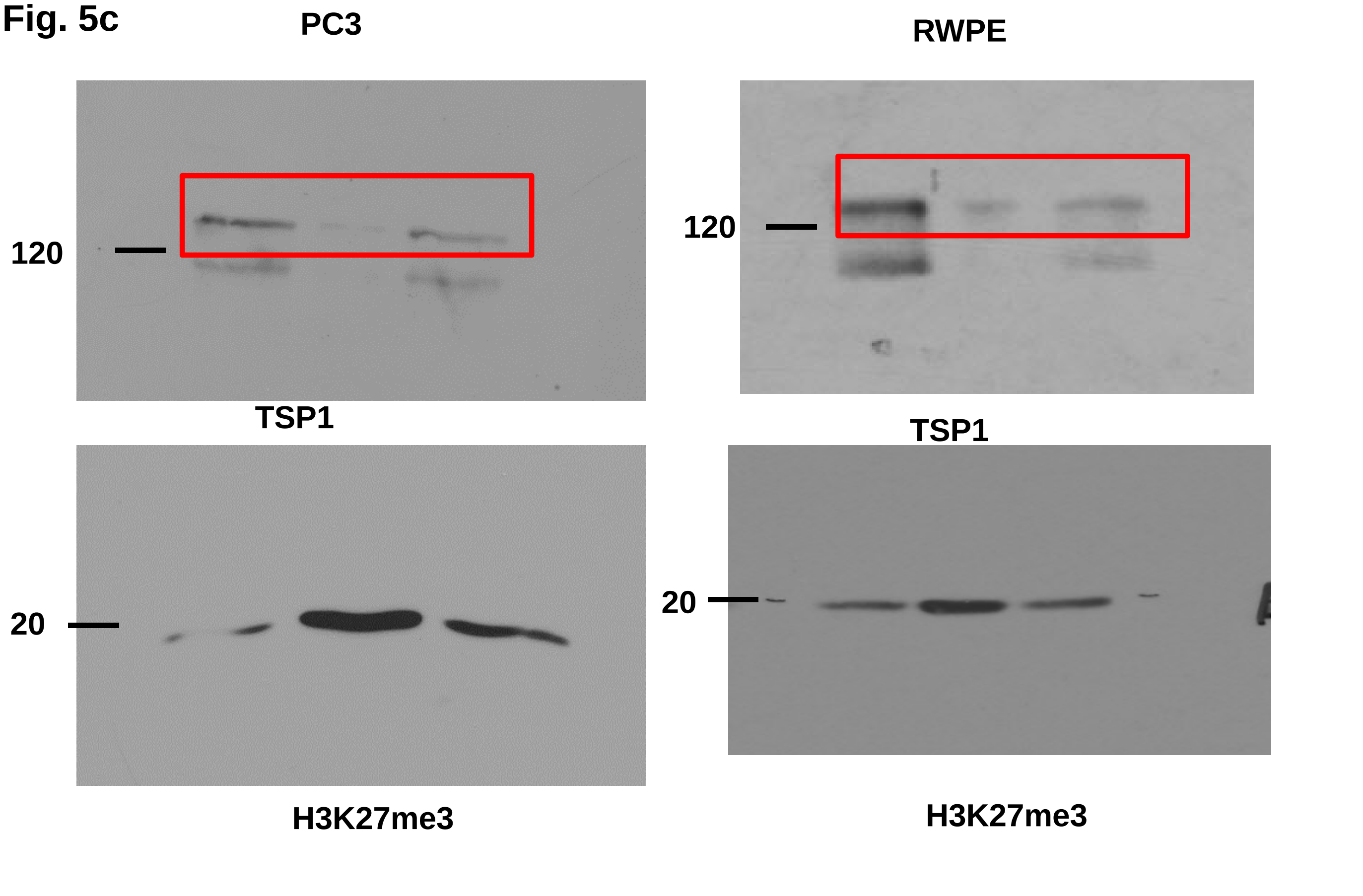
PC3
RWPE
120
120
TSP1
TSP1
20
20
H3K27me3
H3K27me3

Fig. 7a

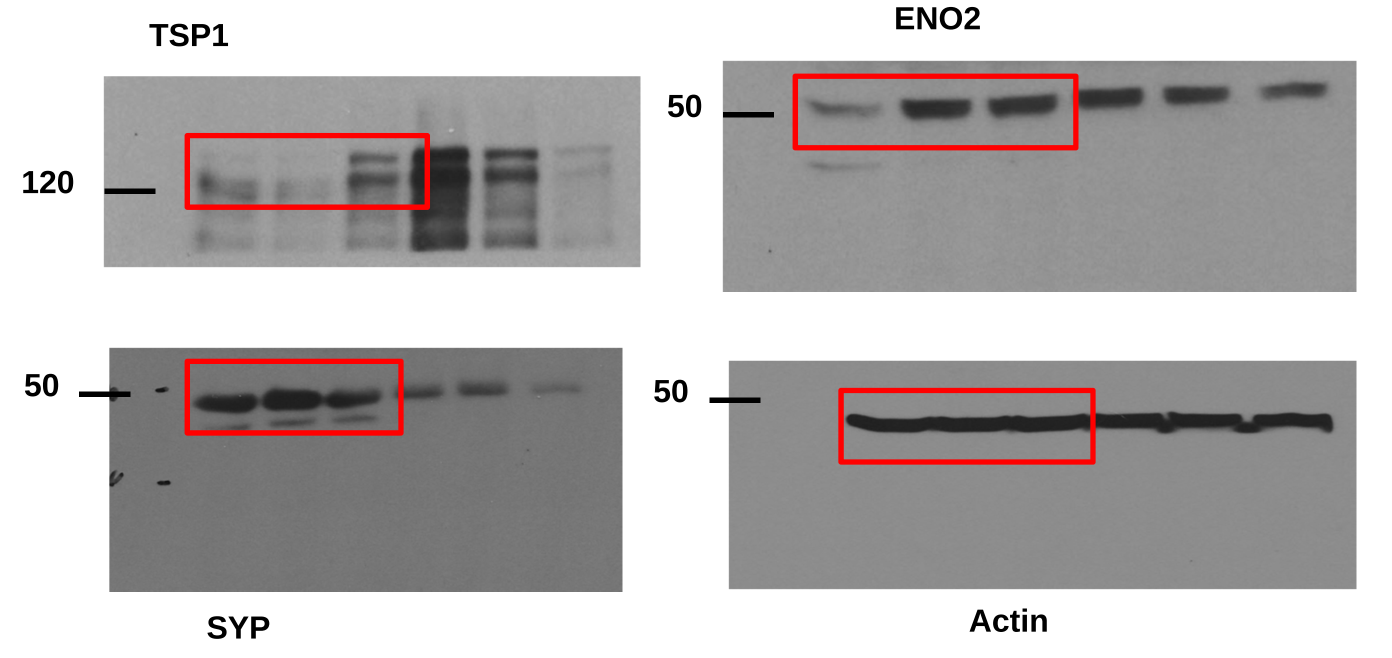
TSP1
ENO2
50
120
50
50
SYP
Actin

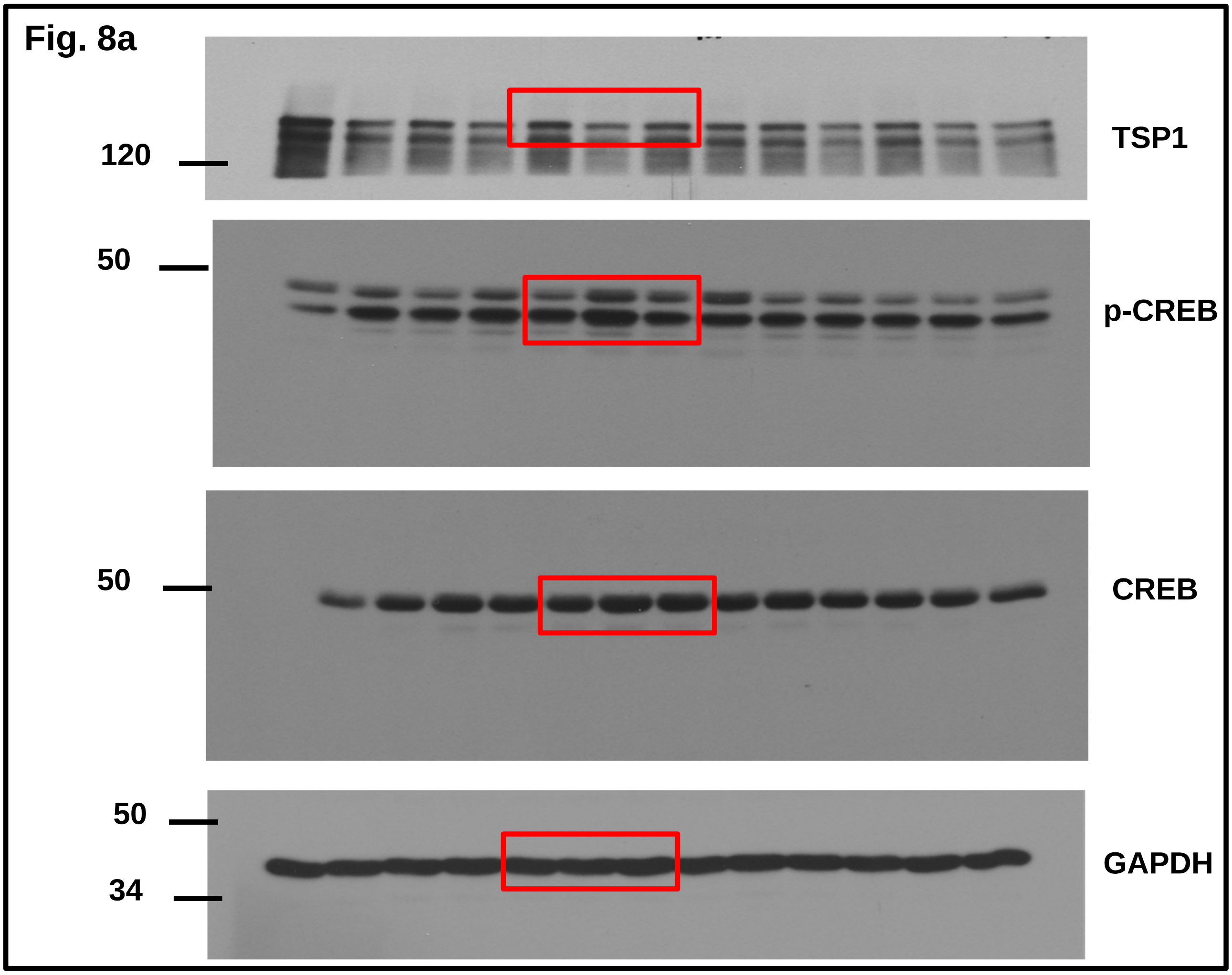
Fig. 8a
120
TSP1
50
p-CREB
50
CREB
50
34
GAPDH

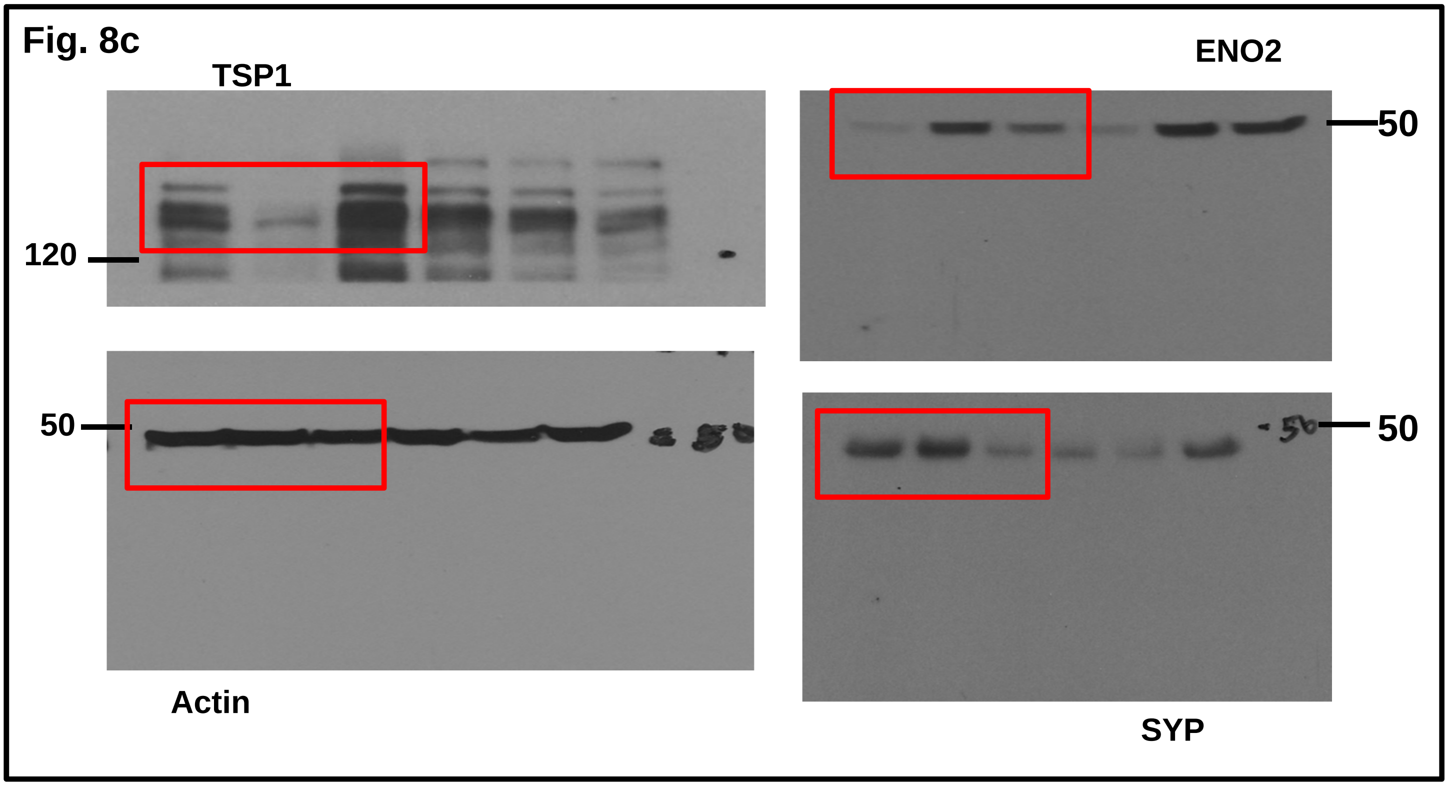
Fig. 8c
TSP1
120
ENO2
50
50
Actin
50
SYP

Fig. 8b

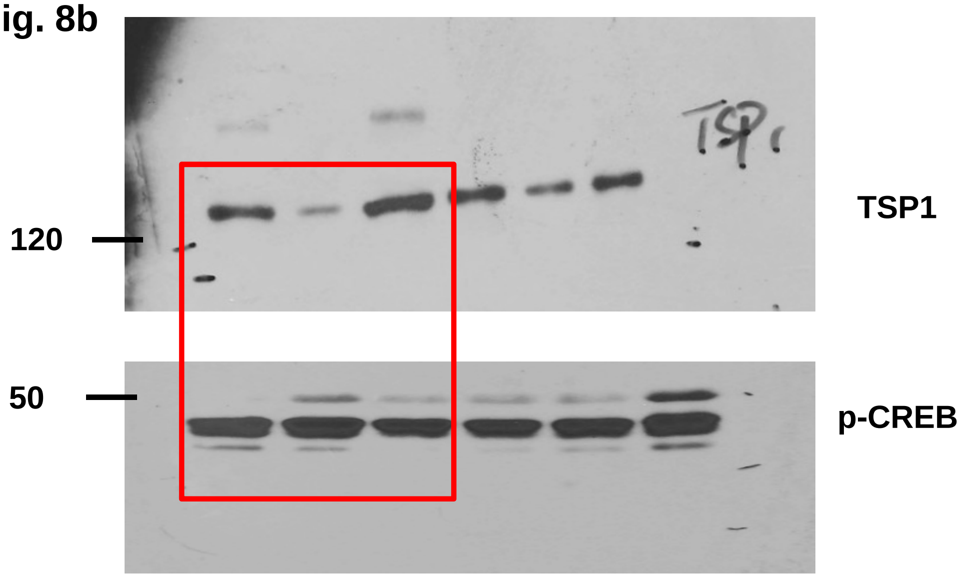

Fig. 8e

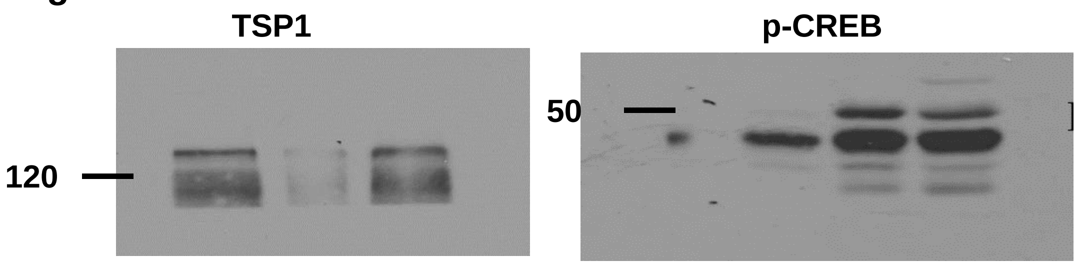

Fig. 8f, 8g

8f 8g

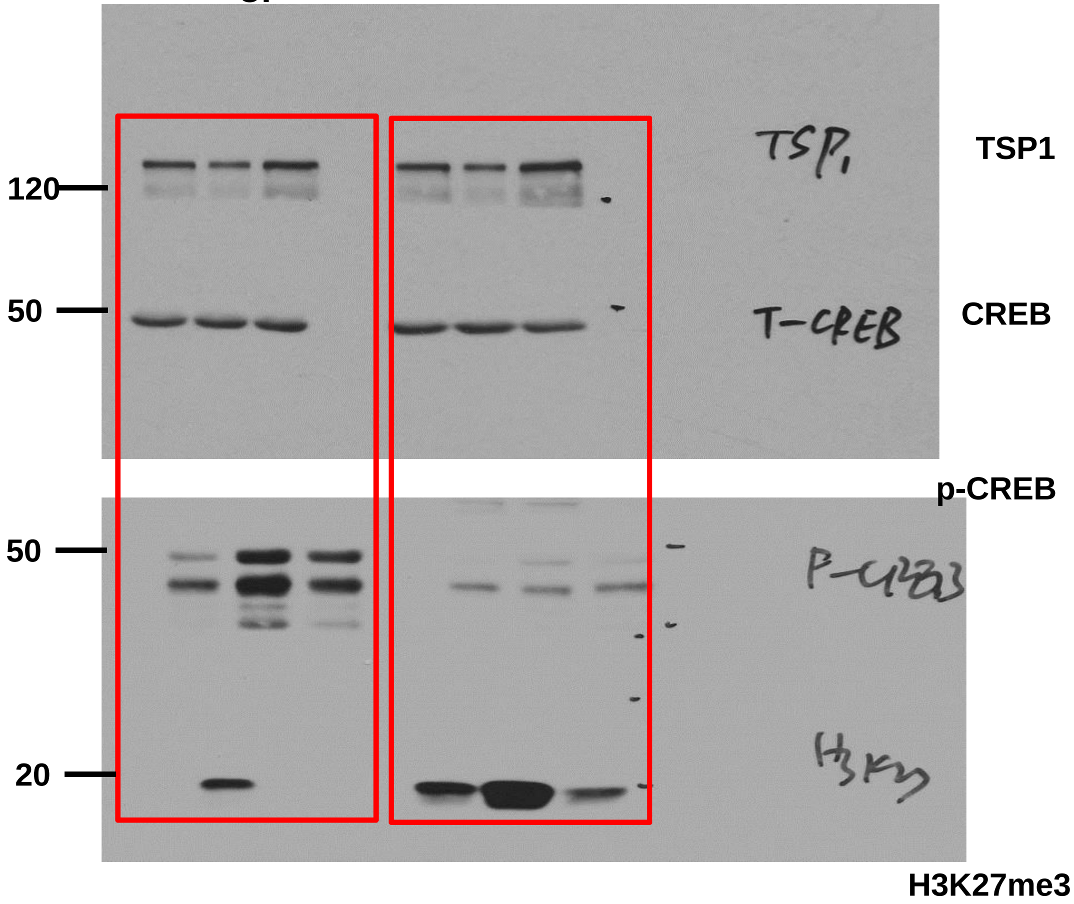

Fig. 9e

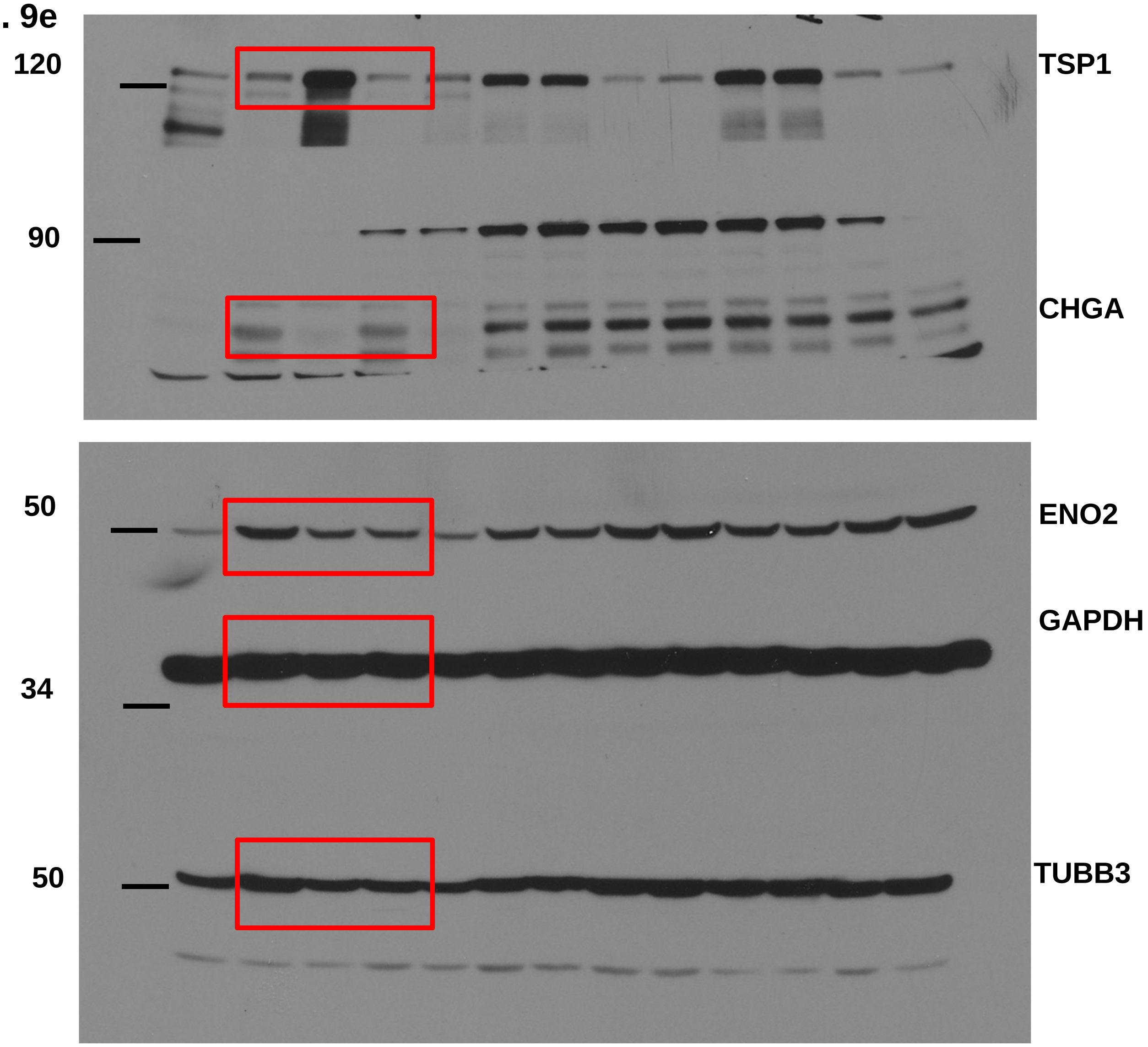

Fig. 10c

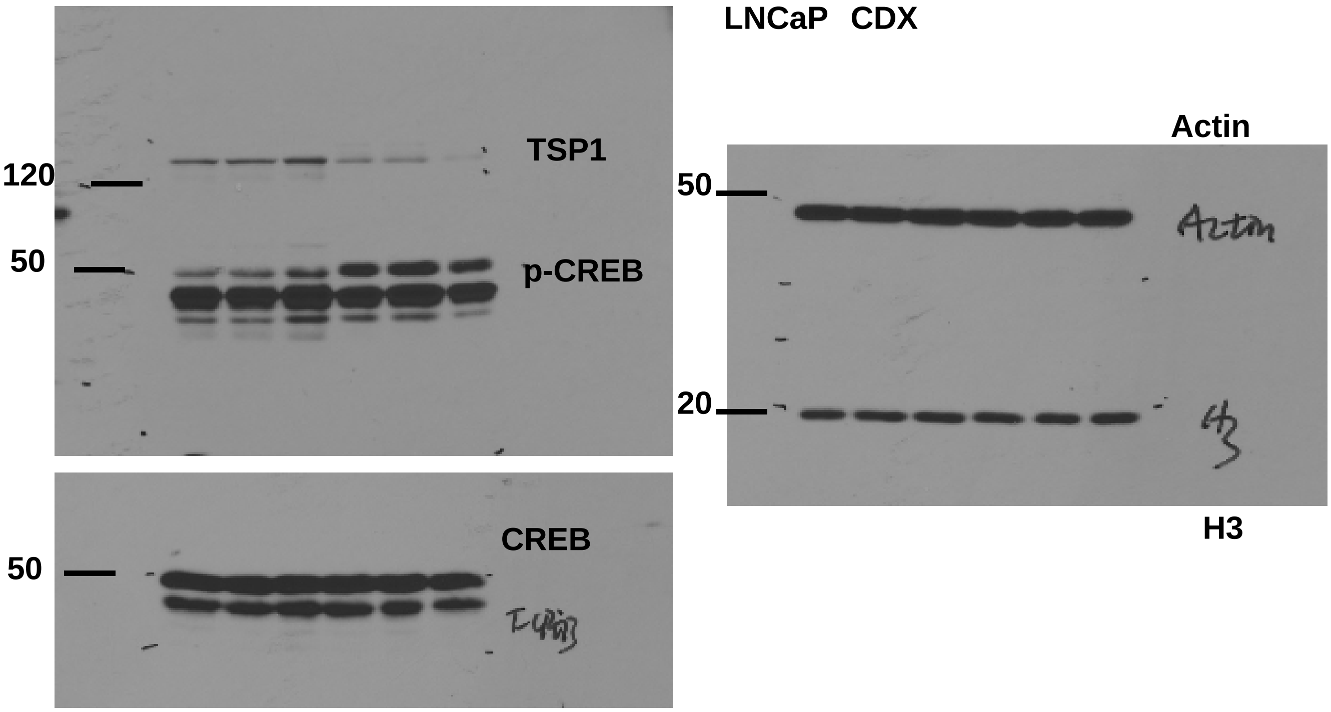

Fig.10c

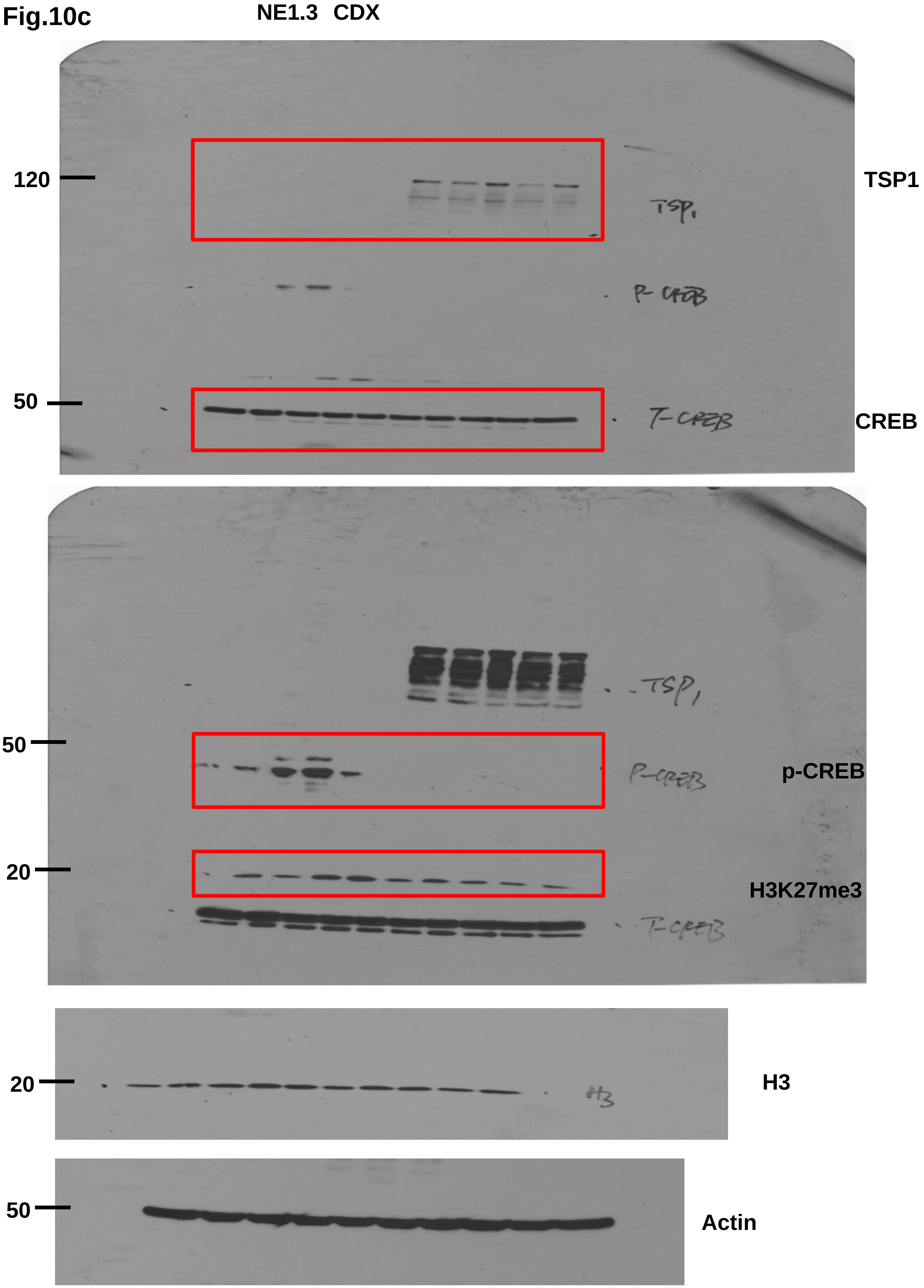

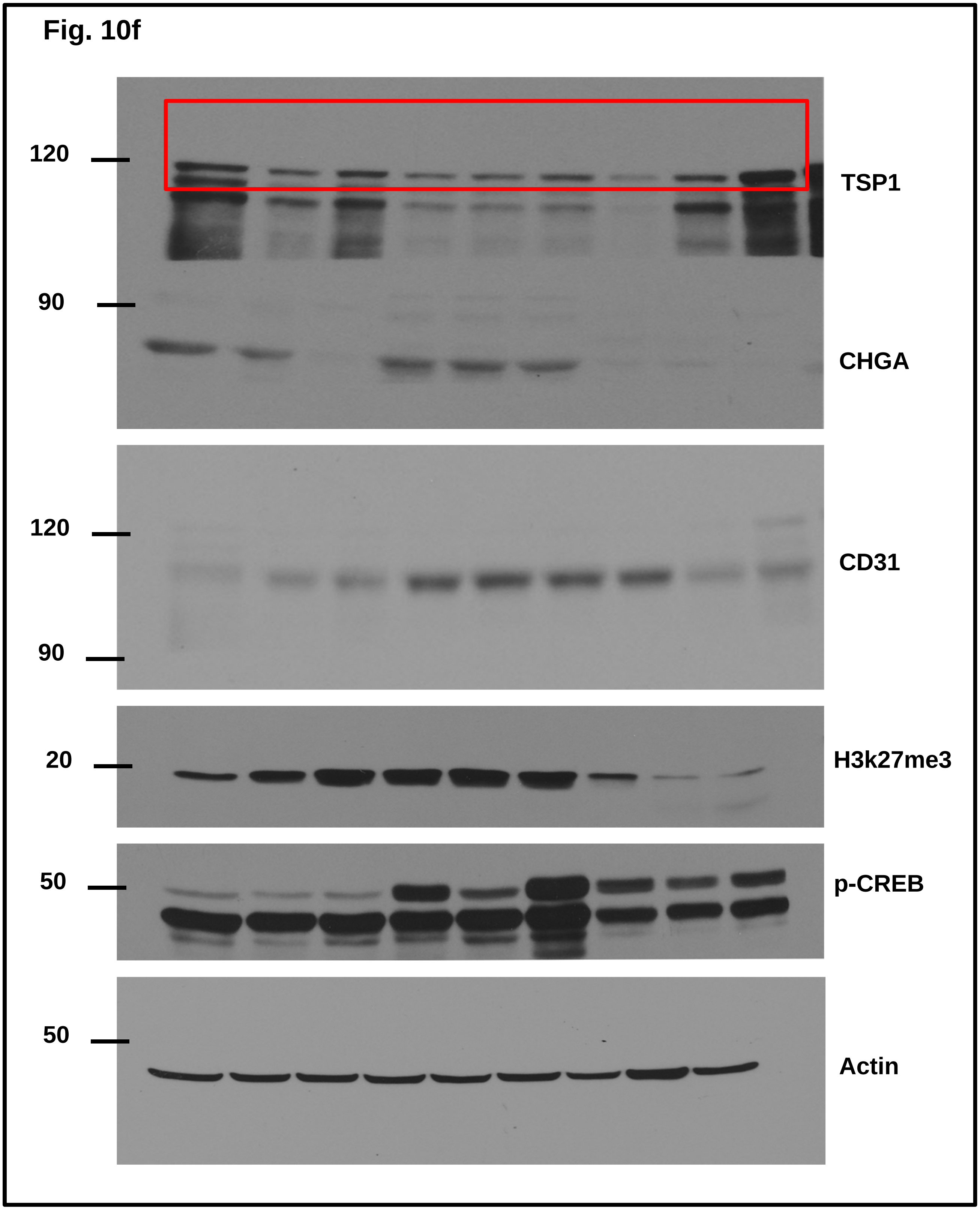

Supplementary Figure 6. The uncropped scans of the most important Western blots

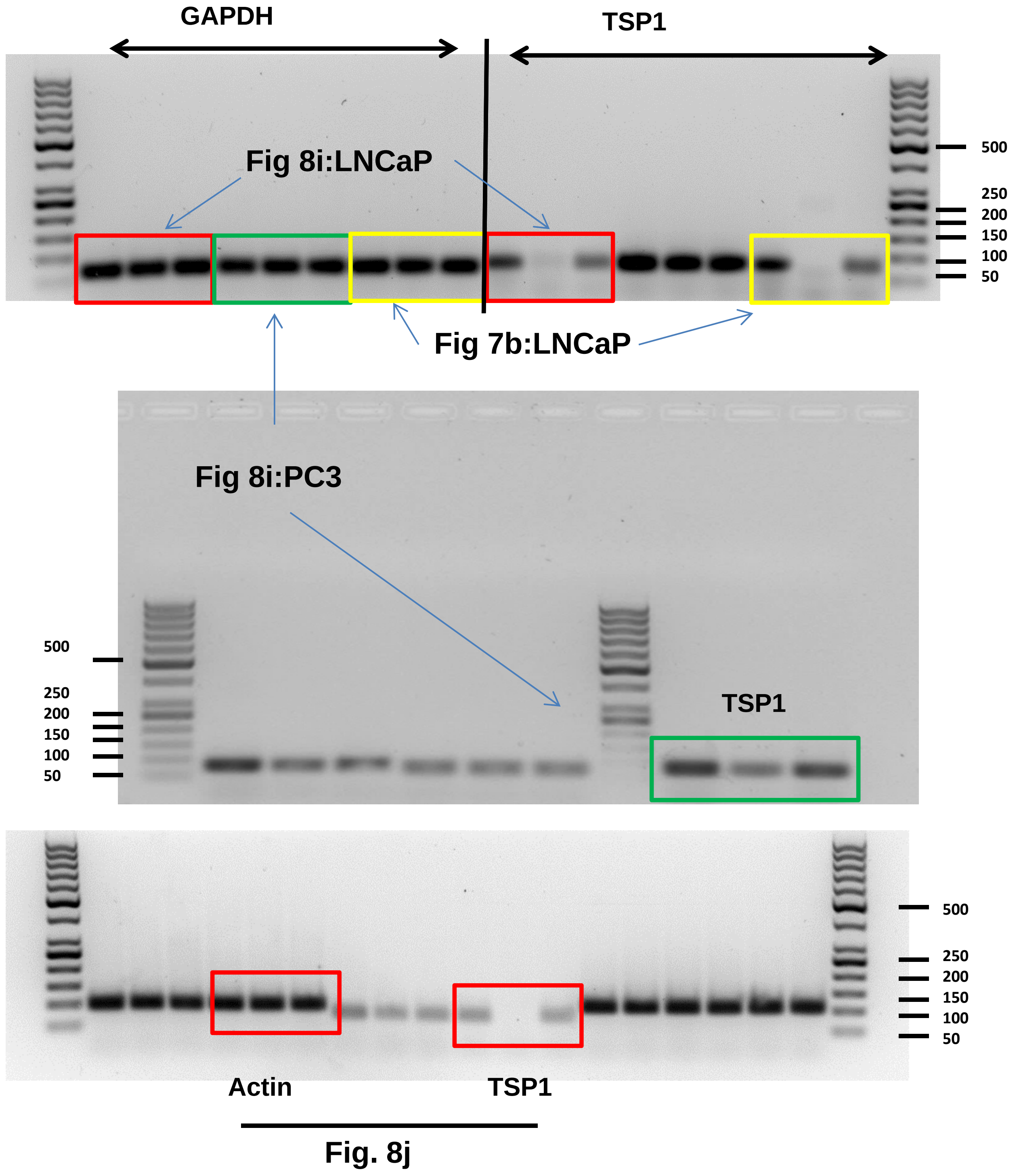

Supplementary Figure 7. The uncropped scans of RT-PCR DNA gel pictures.
